# Trimethylamine‐*N*‐oxide promotes brain aging and cognitive impairment in mice

**DOI:** 10.1111/acel.12768

**Published:** 2018-05-10

**Authors:** Dang Li, Yilang Ke, Rui Zhan, Changjie Liu, Mingming Zhao, Aiping Zeng, Xiaoyun Shi, Liang Ji, Si Cheng, Bing Pan, Lemin Zheng, Huashan Hong

**Affiliations:** ^1^ Department of Geriatrics Fujian Medical University Union Hospital Fuzhou China; ^2^ The Institute of Cardiovascular Sciences and Institute of Systems Biomedicine School of Basic Medical Sciences Peking University Health Science Center Key Laboratory of Molecular Cardiovascular Science Ministry of Education; Key Laboratory of Cardiovascular Molecular Biology and Regulatory Peptides Ministry of Health; Beijing Key Laboratory of Cardiovascular Receptors Research Beijing China; ^3^ Department of Cardiology Fujian Medical University Union Hospital Fuzhou China; ^4^ China National Clinical Research Center for Neurological Diseases Tiantan Hospital The Capital Medical University Beijing China; ^5^ Fujian Medical University Union Hospital Fuzhou China

**Keywords:** brain aging, cognitive function, mammalian target of rapamycin, neuron senescence, oxidative stress, trimethylamine‐*N*‐oxide

## Abstract

Gut microbiota can influence the aging process and may modulate aging‐related changes in cognitive function. Trimethylamine‐*N*‐oxide (TMAO), a metabolite of intestinal flora, has been shown to be closely associated with cardiovascular disease and other diseases. However, the relationship between TMAO and aging, especially brain aging, has not been fully elucidated. To explore the relationship between TMAO and brain aging, we analysed the plasma levels of TMAO in both humans and mice and administered exogenous TMAO to 24‐week‐old senescence‐accelerated prone mouse strain 8 (SAMP8) and age‐matched senescence‐accelerated mouse resistant 1 (SAMR1) mice for 16 weeks. We found that the plasma levels of TMAO increased in both the elderly and the aged mice. Compared with SAMR1‐control mice, SAMP8‐control mice exhibited a brain aging phenotype characterized by more senescent cells in the hippocampal CA3 region and cognitive dysfunction. Surprisingly, TMAO treatment increased the number of senescent cells, which were primarily neurons, and enhanced the mitochondrial impairments and superoxide production. Moreover, we observed that TMAO treatment increased synaptic damage and reduced the expression levels of synaptic plasticity‐related proteins by inhibiting the mTOR signalling pathway, which induces and aggravates aging‐related cognitive dysfunction in SAMR1 and SAMP8 mice, respectively. Our findings suggested that TMAO could induce brain aging and age‐related cognitive dysfunction in SAMR1 mice and aggravate the cerebral aging process of SAMP8 mice, which might provide new insight into the effects of intestinal microbiota on the brain aging process and help to delay senescence by regulating intestinal flora metabolites.

## 1 INTRODUCTION

Human gut microbiota, consisting of trillions of typically nonpathogenic commensal organisms, plays an important role in regulating various biological functions (Lowry et al., 2016). Aging can affect the gut microbiota in terms of composition and functionality. At the same time, there is evidence that loss of gut microbiota diversity can affect the aging process, including brain aging, and may modulate aging‐related changes in cognitive function (Candela, Biagi, Brigidi, O'Toole & De Vos, 2014; Saraswati & Sitaraman, 2015). Recently, trimethylamine‐*N*‐oxide (TMAO), a product of choline, which is metabolized by the gut flora, has attracted increasing interest in medical research. TMAO is involved in a variety of diseases, including cardiovascular events (Tang et al., 2013), atherosclerosis (Koeth et al., 2013), obesity, diabetes (Gao et al., 2014), chronic kidney disease (Missailidis et al., 2016), nonalcoholic liver disease (Chen et al., 2016), Alzheimer's disease (Xu & Wang, 2016), peripheral arterial disease (Senthong et al., 2016), and tumours (Guertin et al., 2017). However, the influence of TMAO in the aging process, including brain aging, has not yet been reported.

Brain aging occurs as individuals become older and is followed by cognitive dysfunction, which affects individuals’ quality of life. Activity‐dependent plasticity of neuronal connections represents the basis of learning and memory—the synaptic plasticity and memory (SPM) hypothesis (Iii, Govindarajan & Tonegawa, 2004; Squire & Davis, 1981). Mammalian target of rapamycin (mTOR) is a multifunctional and highly conserved serine/threonine kinase that has been shown to be a critical integrator of cell signalling in recent years (Sui, Wang & Li, 2008; Tischmeyer et al., 2003). Behavioural experiments have demonstrated that mTOR signalling activity can affect spatial learning, object recognition and inhibitory avoidance memory by regulating synthesis of many synaptic plasticity‐related proteins, including *N*‐methyl‐d‐aspartate receptor subunit 1 (NMDAR1), synaptophysin (SYN) and postsynaptic density‐95 (PSD95) (Gong, Park, Abbassi & Tang, 2006; Schratt, Nigh, Chen, Hu & Greenberg, 2004). This process is mediated by phosphorylation of two major downstream targets of mTOR, p70s6 kinase (p70s6k) and eIF4E‐binding protein (4EBP2) (Garelick & Kennedy, 2011).

Given the close association among TMAO level, gut microbiota and age, we hypothesized that the circulating level of TMAO might be increased with age via the age‐related modulation of the gut microbiota, and TMAO might aggravate the brain aging process. To confirm this hypothesis, we analysed the plasma levels of TMAO in both humans and mice and investigated the effects of TMAO on brain aging and cognitive dysfunction in senescence‐accelerated prone mouse strain 8 (SAMP8) and senescence‐accelerated mouse resistant 1 (SAMR1) mice. Moreover, the possible underlying mechanisms were also examined. We demonstrate that circulating levels of TMAO increased with age in both humans and mice. In addition, TMAO can deteriorate brain aging, probably due to mitochondrial dysfunction and increased oxidative stress. The harmful effects of TMAO on brain aging were due to inhibiting the mTOR signalling pathway and then inducing cognitive dysfunction by destroying chemical synapses and reducing synaptic plasticity.

## 2 RESULTS

### 2.1 The plasma levels of TMAO increased in the aged group in both humans and mice

**Figure 1:**
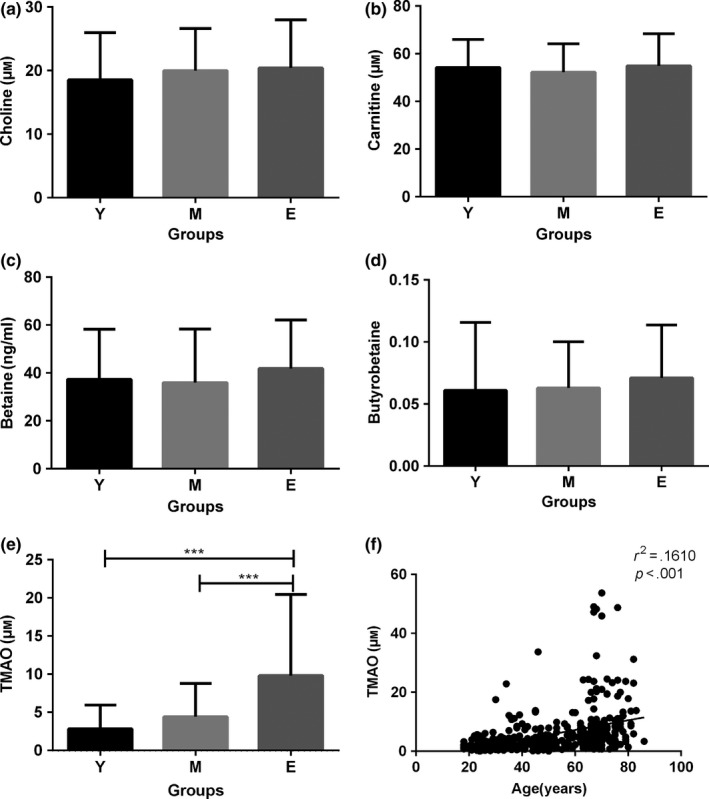
The plasma levels of trimethylamine‐*N*‐oxide (TMAO) increased in the aged group in humans. The plasma was collected from healthy individuals. The levels of plasma TMAO, carnitine, choline, betaine and butyrobetaine were quantified using stable isotope dilution liquid chromatography–tandem mass spectrometry (LC/MS/MS). The levels of plasma choline (a), carnitine (b), betaine (c) and butyrobetaine (d) were unchanged. (e) However, the level of plasma TMAO was significantly higher in the aged group than the young‐ and middle‐aged groups, but there was no significant difference between the young‐ and middle‐aged groups in circulating level of TMAO. (f) Linear regression analysis revealed a tendency that TMAO was positively related to age in the overall studied population. (a–e) Data are shown as the mean ± *SD* (one‐way ANOVA followed by Tukey's multiple comparison test). Y = young adults (*n* = 168), M = middle‐aged adults (*n* = 118), E = elderly adults (*n* = 141) ***p <* *.001

We analysed the plasma TMAO levels using stable isotope dilution liquid chromatography–tandem mass spectrometry (LC/MS/MS) (Tang et al., 2013). The plasma was collected from healthy individuals, and the demographic and clinical data were not significantly different, except age and systolic blood pressure (SBP, Table S1). Several precursors involved in gut microbiota‐dependent conversion of choline to TMAO, such as choline, carnitine, betaine and butyrobetaine, had no significant differences in the three groups (Figure 1a–d). The plasma level of TMAO was significantly higher in the elderly group (*n* = 141, 9.83 ± 10.63 μmol/L) than the young group (*n* = 168, 2.85 ± 3.10 μmol/L) (*p *<* *.01) and the middle‐aged group (*n* = 118, 4.42 ± 4.39 μmol/L) (*p *<* *.01) (Figure 1e). However, there was no significant difference between the young group and the middle‐aged group in circulating level of TMAO. Linear regression analysis revealed a tendency that TMAO was positively related to age in the overall studied population (*r* ^2^ = .1610, *p *<* *.001, Figure 1f).

Moreover, we had compared the plasma TMAO levels in different aged (1‐, 3‐, 6‐ and 10‐month‐old) SAMR1 and SAMP8 mice by LC/MS/MS in our previous study (Ke et al., 2018). We had found an age‐related increase in TMAO levels in both SAMR1 and SAMP8 mice. These results indicated that the plasma levels of TMAO increased in the aged group in both humans and mice.

In this study, we also analysed the plasma TMAO level in SAMR1 and SAMP8 after TMAO treatment for 16 weeks by LC/MS/MS. Compared with the control groups, respectively, the TMAO treatment groups significantly increased plasma TMAO level (*p *<* *.01, Figure S1), which indicated the TMAO treatment could increase circulating level of TMAO effectively.

### 2.2 TMAO impaired cognitive performance of mice

**Figure 2:**
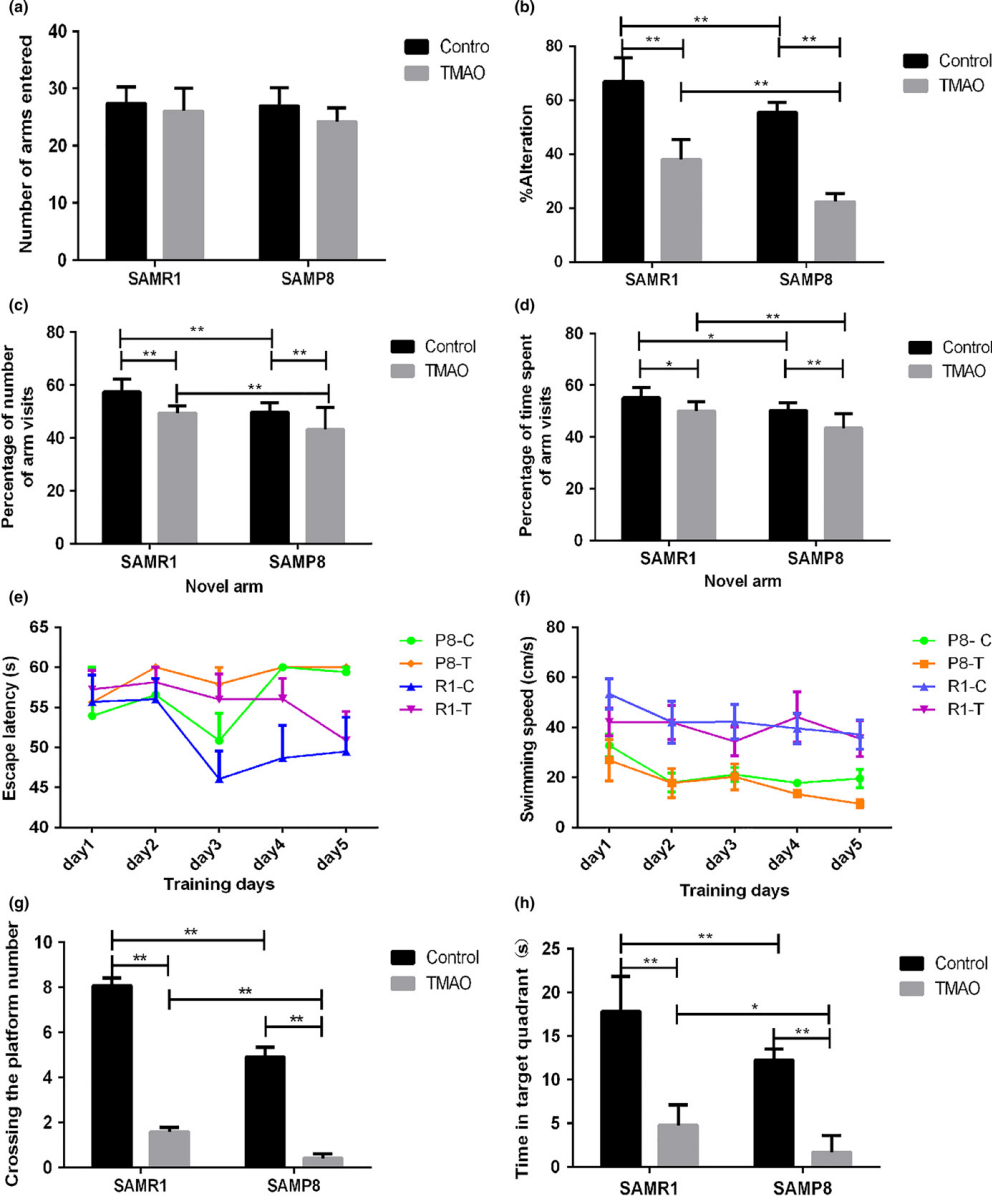
Trimethylamine‐*N*‐oxide (TMAO) impaired cognitive performance of the mice. (a) In the Y‐maze, no significant difference in the total number of arms entered, reflecting the total activity of the animals, was observed based on spontaneous alternation among the four groups. (b) The level of spontaneous alternation was significantly reduced in the TMAO groups compared with the control groups (*p *<* *.01 in R1 groups, *p *<* *.01 in P8 groups). (c and d) In novel object recognition, the percentage of number of visits and the time spent in the novel arm decreased significantly in the TMAO groups compared with the control groups (R1 groups: *p *<* *.01 in entries, *p *<* *.05 in time; P8 groups: *p *<* *.01 in entries, *p *<* *.01 in time), (Figure S2) whereas the data obtained from the start arm and other arm were not significantly different. The P8 groups showed a significant decrease in these indexes compared to the R1 groups. (e) On training days, the escape latency in the TMAO groups was significantly longer than that in the control groups in the Morris water maze test. The P8 groups had a longer escape latency compared with the R1 groups. (f) The swimming speed showed no significant difference among the four groups. (g) The TMAO groups crossed significantly more platforms compared with the control groups (*p* < .01 in R1 groups, *p *<* *.01 in P8 groups). (h) The time spent in the platform quadrant was significantly shorter in the TMAO groups than the control groups (*p *<* *.01 in R1 groups, *p *<* *.01 in P8 groups). (g and h) The P8 groups showed a significant decrease in these indexes compared to the R1 groups. Data are shown as the mean ± SEM. Data from the escape latency and speed in the Morris water maze test were analysed with repeated‐measures two‐way analysis of variance. All other data acquired from behavioural tests were analysed using two‐way ANOVA, and comparisons between two groups were performed using Tukey's multiple comparison test (*n* = 12 each group, *p <* *.05, **p <* *.01)

Aging can be accompanied by cognitive impairment. We tested the effects of TMAO on spatial working memory in mice with the Y‐maze (Kimura, Devi & Ohno, 2010; Ohno et al., 2004) (Figure 2a–d). There was no significant difference among the four groups in the total number of arms entered, which indicated the mice in the four groups had similar working memories (Figure 2a). Nevertheless, there was an apparent decline in the percentage alternation of the TMAO groups compared with the control groups (Figure 2b). Another index of spatial working memory obtained from the trial was novel object recognition, which represents the ability to explore new objects. The results showed that both the percentage of the number of visits and the time spent in the novel arm decreased significantly in the TMAO groups compared with the control groups (Figure 2c and d), whereas the data obtained from start arm and other arm showed no significant differences (Figure S2). The P8 groups showed a significant decrease in these indexes compared with the R1 groups (Figure 2b,c and d).

To further evaluate the effects of TMAO on cognitive performance, we tested the animals in the Morris water maze (Figure 2e–h). Repeated‐measures two‐way analysis, which took the results of two major effects (day and group) into consideration, suggested that the TMAO groups had a significantly increased escape latency compared with the control groups. Moreover, the P8 groups showed a significant increase in the escape latency compared with the R1 groups (Figure 2e). In the first six training days, there was no significant difference among the four groups in the swimming speed (Figure 2f). The results acquired from the “probe test” suggested that the TMAO groups spent less time than the control groups in the quadrant in which the platform was placed before (Figure 2h). At the same time, the number of times the animals crossed over the previous position of the platform was significantly reduced in the TMAO groups compared with their control groups (Figure 2g). As expected, the P8 groups showed a significant decrease in these indexes compared with the R1 groups (Figure 2h and g). These results demonstrated that TMAO could induce and aggravate aging‐related cognitive dysfunction in SAMR1 and SAMP8 mice, respectively.

### 2.3 TMAO induced neuron senescence in the hippocampal CA3 region

**Figure 3:**
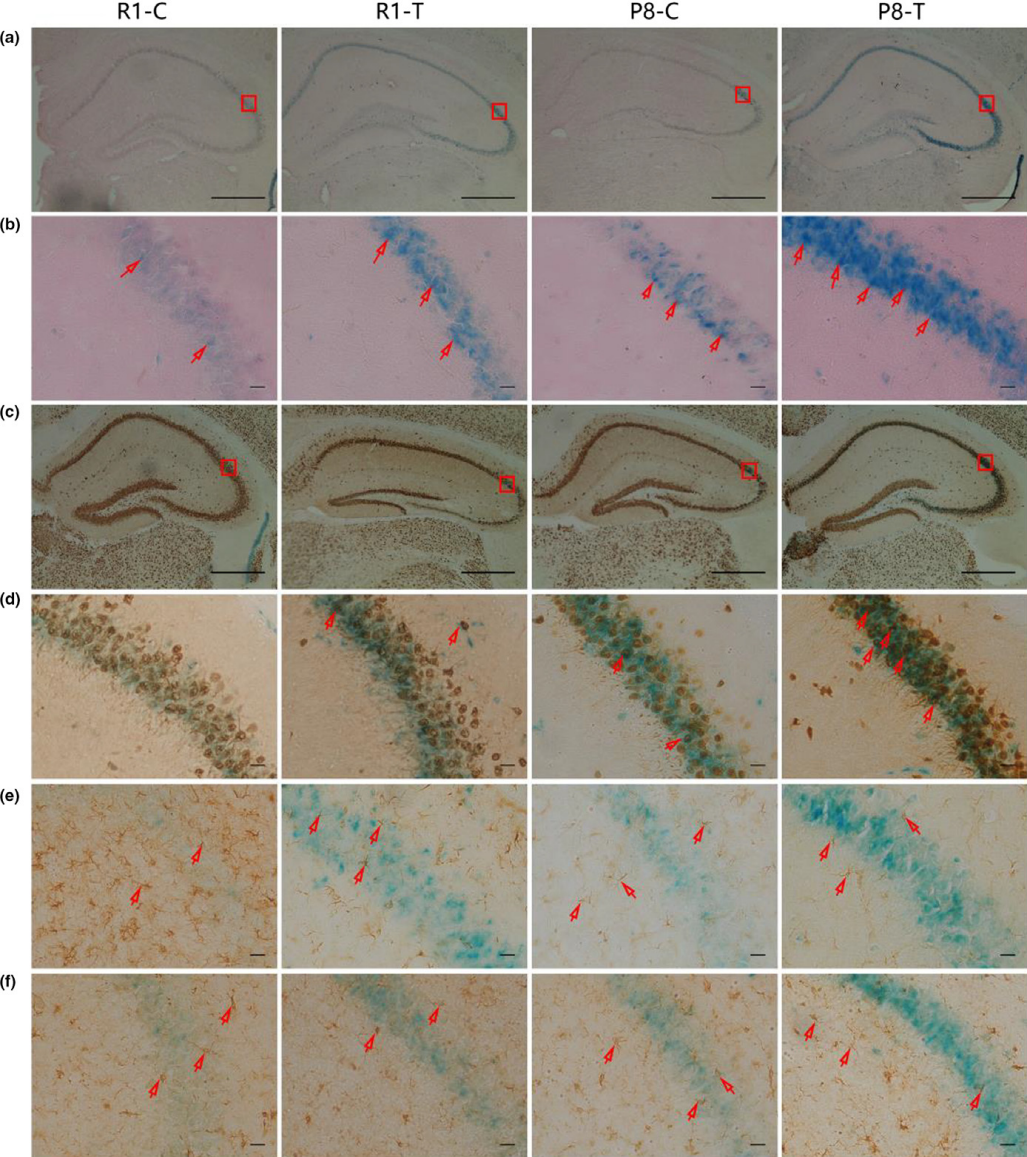
Trimethylamine‐*N*‐oxide (TMAO) induced the aging process in the hippocampal CA3 region. (a and b) SA‐beta‐GAL was detected in the hippocampal CA3 region. The blue cells indicated SA‐beta‐GAL‐positive cells. (c–f) Double staining with SA‐beta‐GAL and immunohistochemical staining for senescent cells. (c and d) Cells positive for SA‐beta‐GAL and NeuN (a marker of neurons, the red arrow indicates senescent neurons) were found in the CA3 region of the hippocampus. (e) Cells negative for SA‐beta‐GAL but positive for GFAP (a marker of astrocytes, the red arrow indicates astrocytes) were found in the CA3 region of the hippocampus. (f) Cells negative for SA‐beta‐GAL but positive for Iba1/AIF1 (a marker of microglia, the red arrow indicates microglia) were found in the CA3 region of the hippocampus. These results suggested that TMAO could promote cell senescence in the hippocampal CA3 region, and the senescent cells were primarily neurons (*n* = 6 each group, scale bar: b, d, e, f = 100 μm, a, c = 50 μm)

Senescence‐associated beta‐galactosidase (SA‐beta‐GAL) staining has become one of the most commonly used markers of cellular senescence. Consistent with the previous findings, SA‐beta‐GAL‐positive cells were observed in the hippocampal CA3 region (Geng, Guan, Xu & Fu, 2010). R1‐C mice showed a few SA‐beta‐GAL‐positive cells, whereas the R1‐T and P8‐C groups had more cells showing prominent SA‐beta‐GAL staining. As expected, the P8‐T group had the most cells showing robust SA‐beta‐GAL staining among all groups (Figure 3a and b).

To further identify the class of the SA‐beta‐GAL‐positive cells, we stained the sections for markers of neurons, astrocytes and microglia using immunohistochemical staining after SA‐beta‐GAL staining (double staining). We found cells positive for both SA‐beta‐GAL and NeuN (a marker of neurons, the red arrow indicates senescent neurons, Figure 3c and d) and cells negative for SA‐beta‐GAL but positive for both GFAP (a marker of astrocytes, the red arrow indicates astrocytes, Figure 3e) and Iba1/AIF1 (a marker of microglia, the red arrow indicates microglia, Figure 3f) in the CA3 region of the hippocampus. The results suggested that TMAO could promote cell senescence in the hippocampal CA3 region, and the senescent cells were primarily neurons. Our findings suggested that TMAO could induce brain aging in SAMR1 and aggravate the cerebral aging process of SAMP8.

### 2.4 TMAO damaged the ultrastructure of the hippocampal CA1 region

**Figure 4:**
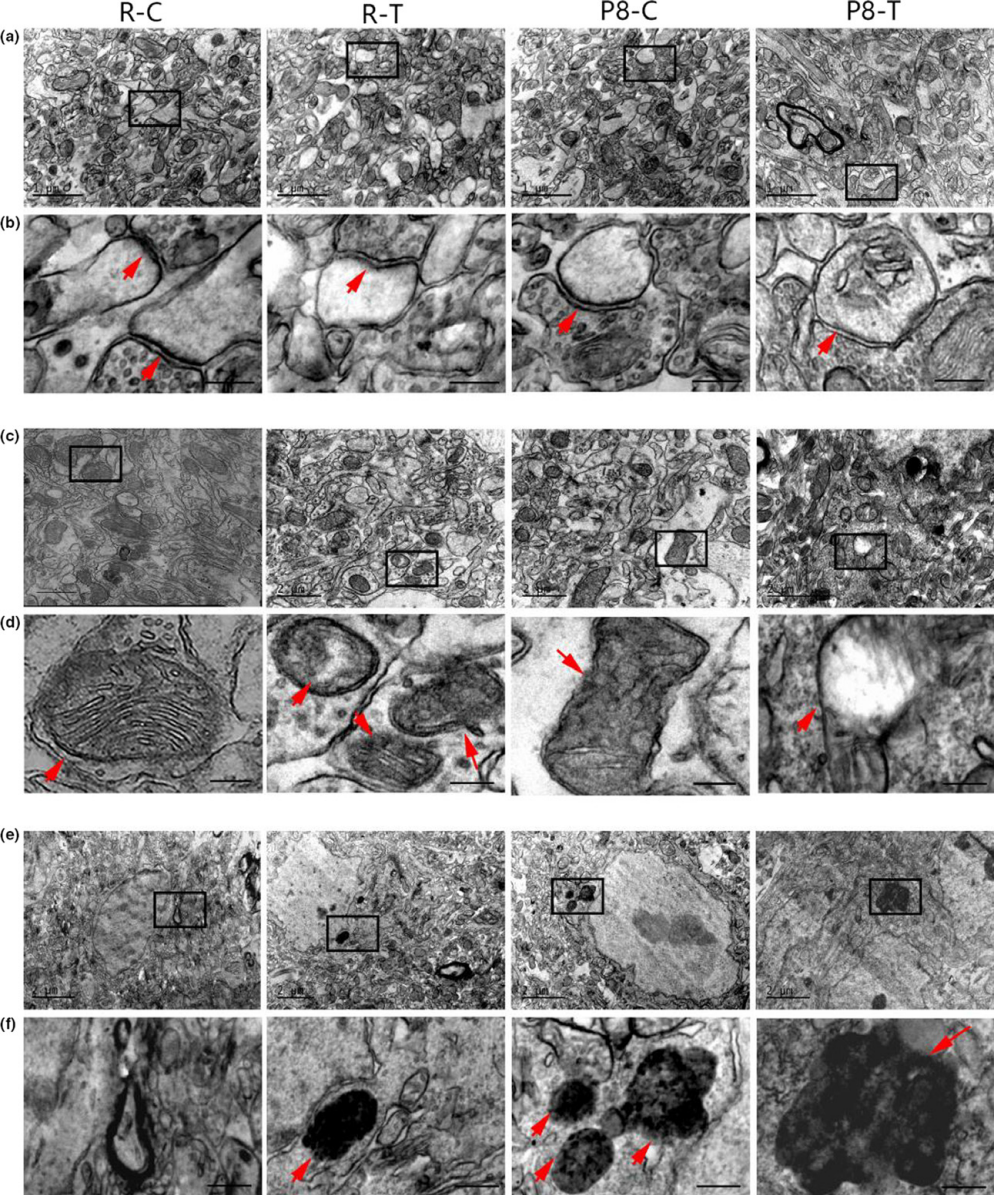
Trimethylamine‐*N*‐oxide (TMAO) damaged the ultrastructure of the hippocampal CA1 region. (a and b, the red arrow indicates chemical synapses) Chemical synapses in R1‐C appeared normal: abundant synaptic vesicles were inside the presynaptic membrane. Postsynaptic density was concentrated on the intracellular surface of the opposing postsynaptic membrane. The synaptic cleft appeared narrow and rigidly paralleled with membranes. The structure of synapses in the TMAO groups was damaged compared with that in the control groups, with fewer synaptic vesicles, reduced postsynaptic area, a lower concentration of electron‐dense substances and a wider synaptic cleft. P8‐T showed more damage with a sharply decreased concentration of electron‐dense substances, severely decreased synaptic vesicles and postsynaptic area, obvious widening of the synaptic cleft and swelling of the postsynaptic membrane. (c and d, e and f, the red arrow indicates mitochondria and lipofuscin, respectively) The ultrastructure of cells in the R1‐C group appeared normal; the cells contained abundant normal mitochondria, and little lipofuscin was observed. The structure of cells in the TMAO groups was damaged compared with that of the control groups, with deformed mitochondria, reduced mitochondrial cristae and abundant lipofuscin, especially in the P8‐T group (*n* = 6 each group, scale bar: a = 1 μm, b = 200 nm, c, e = 2 μm, d, f = 400 nm)

The ultrastructure of chemical synapses in the hippocampal CA1 region was observed with a transmission electron microscope (TEM) (Figure 4a and b). Chemical synapses of the mice in R1‐C appeared normal compared with those in P8‐C. However, chemical synapses of the mice in the TMAO groups showed more damage compared with those of their control groups. There were fewer synaptic vesicles, a reduced postsynaptic area, a lower density of electron‐dense substances and a wider synaptic cleft in the TMAO groups compared to the control groups. Moreover, compared with R1‐T, P8‐T showed more damage in the anterior region, including a sharply decreased density of electron‐dense substances, severely decreased synaptic vesicles and postsynaptic area, widening of the synaptic cleft and swelling of the postsynaptic membrane.

As shown in Figure 4c–f, the ultrastructure of cells in the hippocampal CA1 region appeared irregular in the TMAO groups. The mitochondria and endoplasmic reticulum appeared swollen, some mitochondrial cristae were damaged and reduced, and some lipofuscin accumulation was observed in the cells. Furthermore, the cells from P8‐T were severely damaged compared with those of R1‐T. However, the cells in R1‐C appeared normal compared with those of the other groups.

### 2.5 TMAO increased the level of oxidative stress in the hippocampus of mice

**Figure 5:**
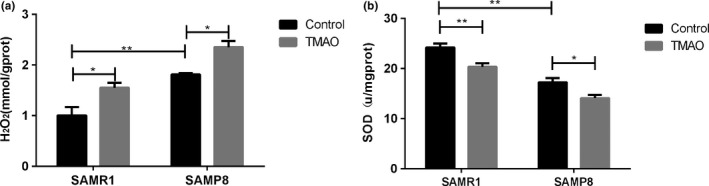
Trimethylamine‐*N*‐oxide (TMAO) increased the level of oxidative stress in the hippocampus of the mice. (a) TMAO significantly increased the concentration of H_2_O_2_. (b) TMAO significantly inhibited the total SOD activity. Data are shown as the mean ± SEM (*n* = 6 each group, two‐way ANOVA, comparisons between two groups were performed using Tukey's multiple comparison test. *p < .05, **p <* *.01)

Aging can be attributed to increasing oxidative stress. To explore the possible mechanism involved in TMAO‐promoted brain aging, we evaluated the levels of hydrogen peroxide (H_2_O_2_) and total superoxide dismutase (T‐SOD) activity in the hippocampus. H_2_O_2_ was lower in R1‐C than in P8‐C. However, it was significantly increased in the TMAO groups compared with the control groups, especially in P8‐T (Figure 5a). Moreover, the activity of T‐SOD was greater in R1‐C than P8‐C. However, a significant decline could be detected in the TMAO groups (Figure 5b).

### 2.6 TMAO reduced the expressions of synaptic plasticity‐related proteins

**Figure 6:**
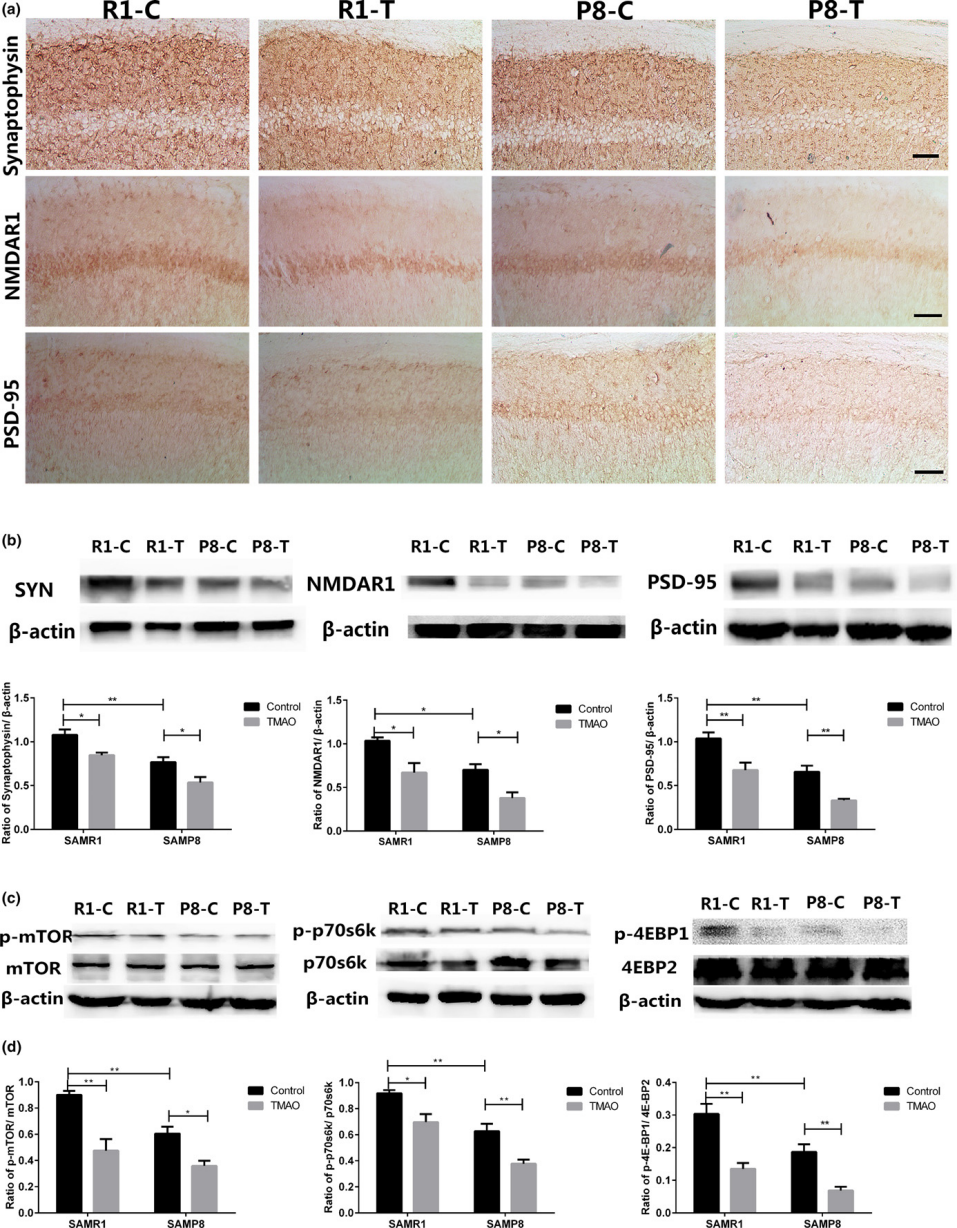
Trimethylamine‐*N*‐oxide (TMAO) reduced the expression of synaptic plasticity‐related proteins by down‐regulating the activity of the mTOR signalling pathway. (a) Synaptophysin (SYN), postsynaptic density 95 (PSD‐95) and *N*‐methyl‐d‐aspartate 1 (NMDAR1) were shown by immunohistochemical staining in the hippocampus. The expression levels of SYN, NMDAR1 and PSD‐95 were significantly decreased in the P8‐C group compared with the R1‐C group. There was a significant decrease in the expression of these synaptic‐associated proteins in the TMAO groups compared with their control groups (scale bar = 50 μm). (b) The expression levels of SYN, NMDAR1 and PSD‐95 were assayed by Western blotting. To examine the possible mechanism involved in the decline of synaptic‐associated proteins, three proteins (mTOR, p70s6k and 4EBP2) of the mTOR signalling pathway were assessed by RT–PCR and Western blotting. (Figure S3A) The mRNA levels of mTOR, p70s6k and 4EBP2 were not significantly different in the groups assessed by RT–PCR. (c) Phosphor‐mTOR/mTOR, phosphor‐p70s6k/p70s6k and phosphor‐4EBP1/4EBP2 were monitored by Western blotting. β‐Actin was used as a loading control. The bands in the Western blotting were scanned, and the ratios of optical density of specific bands and β‐actin are illustrated. TMAO inhibited the active form of mTOR, p70s6k and 4EBP2 (phosphorylated). Data are shown as the mean ± SEM (*n* = 6 each group, two‐way ANOVA, comparisons between two groups were made using Tukey's multiple comparison test. **p <* *.01). The samples derived from the same experiment and the blots were processed in parallel

Synaptic plasticity‐related proteins are a key component of the learning machinery in the brain (Iii et al., 2004). The expression levels of SYN, PSD‐95 and NMDAR1 were significantly decreased in P8‐C compared with R1‐C. Moreover, the TMAO groups showed an apparent decline compared with the control groups, especially in P8‐T (Figure 6a). Similar results were obtained from the Western blotting analyses of these three synaptic plasticity‐related proteins (Figure 6b). These results suggested that TMAO could decrease the expression levels of these synaptic plasticity‐related proteins.

### 2.7 TMAO down‐regulated the activity of the mTOR signalling pathway

The mTOR signalling pathway modulates the synthesis of proteins involved in learning and memory (Schratt et al., 2004; Wullschleger, Loewith & Hall, 2006). To explore the possible mechanism involved in the reduction in synaptic plasticity‐related proteins, we used qRT–PCR and Western blotting to evaluate the activity of the mTOR signalling pathway. The phosphorylation levels of mTOR, p70s6k and 4EBP2 were significantly decreased in P8‐C compared with R1‐C. These proteins were decreased significantly in the TMAO groups compared to the control groups, especially in P8‐T (Figure 6c and d). However, the mRNA levels and expression levels of the total proteins were not significantly different in these groups (Figure S3).

The novel findings of our research included the following: (i) the circulating levels of TMAO increased with age in both humans and mice; (ii) compared with age‐matched SAMR1‐control mice, SAMP8‐control mice exhibited a brain aging phenotype characterized by more senescent cells in the hippocampal CA3 region and cognitive dysfunction; (iii) TMAO treatment increased the number of senescent cells, which were primarily neurons in the hippocampal CA3 region of the mice; (iv) more mitochondrial impairments and increased superoxide production were detected in the TMAO‐treated mice; (v) TMAO could increase synaptic damage and reduce the expression levels of both synaptic plasticity‐related proteins and the mTOR signalling pathway; (vi) TMAO‐treated mice showed substantial cognitive impairment; (vii) the harmful effects of TMAO on mouse brains were more serious in SAMP8 mice than SAMR1 mice. Taken together, the above results indicate that the plasma TMAO level is associated with aging and can increase mitochondrial impairments driven by oxidative stress and reduce the expression levels of synaptic plasticity‐related proteins by inhibiting the mTOR signalling pathway. These changes result in neuron senescence and induce and deteriorate brain aging and age‐related cognitive dysfunction in SAMR1 and SAMP8.

## 3 DISCUSSION

Previous studies have emphasized the influence of gut microbiota in the development of many diseases (Adnan et al., 2017; Tang & Hazen, 2014). TMAO, a metabolite of intestinal flora, was shown to be closely associated with age‐related diseases. The level of plasma TMAO can be influenced by several factors (Romano, Vivas, Amadornoguez & Rey, 2015), including gut microbiota, diet, liver FMO enzymes and kidney function. Previous studies have shown that aging can alter the structure and function of intestinal flora (O'Toole & Jeffery, 2015), especially in elderly people (>65 years of age) (Claesson et al., 2012). Thus, the TMAO plasma level may be altered during the aging process. In our recently published study, we found that the circulating TMAO level was markedly higher in SAMP8 mice than age‐matched SAMR1 mice (Ke et al., 2018). Compared with age‐matched SAMR1 mice, SAMP8 mice are more prone to aging characteristics (Flood & Morley, 1997). Our findings were consistent with the results of previous studies showing that the circulating level of TMAO was increased with age in rats (Li, Chen, Gua & Li, 2017). In the current study, we found that the plasma level of TMAO increased with age in humans (Figure 1e). These results suggested that the plasma level of TMAO could increase during the aging process. Thus, TMAO may play a role in the development of age‐related diseases.

Increasing evidence has shown that the action of microbial metabolites can be a link between external factors and central nervous system function and behaviour (Cryan & Dinan, 2012; Hsiao et al., 2013). However, the exact mechanisms underlying how microbial metabolites regulate brain function have not been fully elucidated. A recent study proposed that TMAO is highly associated with Alzheimer's disease, which provides new insight into the influence of microbial metabolites on brain aging and cognitive function (Xu & Wang, 2016). In our research, we observed that TMAO could induce neuron senescence (Figure 3c and d) and destroy mitochondria in the hippocampal CA3 region of mice (Figure 4c and d), leading to aggravated brain aging in mice. Recent studies demonstrated that elevating the circulating TMAO level could impair mitochondria and induce cardiovascular events (Xu & Wang, 2016). Mitochondrial energy metabolism impairments can decrease ATP production and increase calcium buffer damage and H_2_O_2_ generation, which is considered a crucial player in both aging and neurodegenerative disorders (Johri, Chandra & Beal, 2013). In our study, increased levels of H_2_O_2_ were found in the TMAO‐treated groups (Figure 5a), which could potentially reflect increased mitochondrial free radical production. Thus, we speculated that TMAO could deteriorate brain aging, probably due to mitochondrial dysfunction driven by increased oxidative stress.

Cognitive deficit and learning impairment, accompanied by brain aging, result from changes in synaptic plasticity and reduced synthesis of synaptic plasticity‐associated proteins in the hippocampus. Through TEM, we observed that TMAO could induce synaptic loss and deterioration of synaptic plasticity in the hippocampal CA1 area of the mice (Figure 4). Furthermore we examined the synthesis of synaptic plasticity‐related proteins (Figure 6). Located in the synaptic vesicles, synaptophysin is believed to be a good indicator of synaptic density and synaptic formation (Alladi, Wadhwa & Singh, 2002; Ozçelik et al., 1990). Previous studies on brain aging and neurodegenerative diseases have illustrated the relationship between loss of SYN and cognitive deficit (Heinonen et al., 1995; Joca, Guimarães & Delbel, 2007). Because of its role in long‐term potentiation (LTP), NMDAR1 is believed to strengthen synaptic plasticity, which is considered the molecular basis of learning and memory (Lau, Saha, Faris & Russek, 2004). PSD‐95 is a core component of postsynaptic density (PSD) and is thought to be important in the control of excitatory synapse function (Béïque et al., 2006; Vickers et al., 2006). Reduced expression of PSD‐95 induces cognitive and memory dysfunction (Migaud et al., 1998). Our data from both immunohistochemical staining and Western blotting suggested that TMAO could down‐regulate the expression levels of synaptic plasticity‐related proteins, such as SYN, NMDAR1 and PSD‐95 (Figure 6), which caused cognitive impairment in mice.

The mTOR signalling pathway is important in neuronal development, synaptic plasticity and long‐term potentiation (LTP) by phosphorylating its two downstream substrates, p70s6k and 4EBP2. Studies showed that rapamycin inhibited mTOR signalling activity by binding and interacting with the FK506‐binding protein FKBP12, which reduced long‐term memory in mice (Sui et al., 2008; Tischmeyer et al., 2003). Evidence has confirmed that ginsenoside Rg1 improved age‐related cognitive deficit by up‐regulating the expression levels of the mTOR signalling pathway (Yang, Zhang, Zheng, Shen & Chen, 2014). Thus, the mTOR signalling pathway can serve as a key indicator in our research to explore the effects of TMAO on cognitive decline and learning impairment. In our current study, we found that the expression levels of the synaptic plasticity‐related proteins (SYN, NMDAR1 and PSD‐95, Figure 6a and b) and the mTOR signalling pathway were down‐regulated in the TMAO‐treated mice (Figure 6c and d).

In conclusion, our results indicate that TMAO is associated with aging, especially brain aging, in mice. Moreover, TMAO promotes neuron senescence, damages synapses, down‐regulates the expression levels of both synaptic plasticity‐related proteins and the mTOR signalling pathway and therefore deteriorates brain aging and cognitive decline in mice. Our findings may provide new insight into the effect of intestinal microbiota on brain aging and age‐related cognitive dysfunction and help to delay senescence by regulating intestinal flora metabolites.

## 4 EXPERIMENTAL PROCEDURES

### 4.1 Human subjects

Randomly selected subjects were recruited from healthy individuals who came to Fujian Medical University Union Hospital for routine physical examination. Subjects with high blood pressure, stroke, cardiovascular disease, hyperglycaemia, dementia, abnormal hepatic and renal function and acute diseases were excluded from this study. All 427 blood donors were grouped by age: the young adults (age range 18–44 years, *n* = 168), the middle‐aged adults (age range 45–64 years, *n* = 118) and the elderly (above 65 years, *n* = 141). All examinations were performed with the informed consent of the subjects. The protocol of the study was conducted in accordance with the Declaration of Helsinki and was approved by the Ethics Committee of Fujian Medical University Union Hospital (Fuzhou, Fujian, China).

Blood was drawn into pyrogen‐free tubes with EDTA after the subjects fasted for 12 hr and was centrifuged (12,000 *g* for 15 min). Serum samples were stored at −80°C for TMAO measurement. Other related information was listed in the Data S1.

### 4.2 Measurement of plasma TMAO in humans

The TMAO plasma level was measured using liquid chromatography coupled with triple quadrupole mass spectrometry as described previously (Tang et al., 2013). Briefly, 20 μl of plasma was aliquoted to a 1.5 ml Axygen tube and mixed with 80 μl of 10 μm internal standard comprised of d9‐TMAO in methanol. The protein in the samples was precipitated, and supernatants (70 μl) were analysed by injection onto a silica column (2.0 × 150 mm, Luna 5 μ Silica 100A; Cat. No. 00F‐4274‐B0, Phenomenex, Torrance, CA) at a flow rate of 0.4 ml/min using a LC‐20AD Shimadzu pump system and SIL‐20AXR autosampler interfaced with an API 5500Q‐TRAP mass spectrometer (AB SCIEX, Framingham, MA). A discontinuous gradient was generated to resolve the analytes by mixing solvent A (0.1% propanoic acid in water) with solvent B (0.1% acetic acid in methanol) at different ratios starting from 2% B, which was linearly increased to 95% B over 5.0 min, followed by a hold for 1.0 min, and then a return back to 2% B. Analytes were monitored using electrospray ionization in positive‐ion mode with multiple reaction monitoring (MRM) of precursor and characteristic production transitions of TMAO at m/z 76→58, d9‐TMAO at m/z 85→66, choline at m/z 104→59.8, d9‐choline at m/z 113.2→68.9, carnitine at m/z 162.1→103, d9‐carnitine at m/z 171.1→102.8, betaine at m/z 118→59 and d11‐betaine at 129.1→65.9. A standard curve was generated to obtain the precise concentration of the three analytes. Then, 20 μl of various concentration standards (0–100 μm) was processed with the same procedure. Standard curves were acceptable when the coefficient of determination (*R* ^2^) reached 0.999.

### 4.3 Animals

Twenty‐four‐week‐old male, specific pathogen‐free (SPF) SAMR1 (senescence‐accelerated mouse resistant 1) and SAMP8 (senescence‐accelerated prone mouse strain 8) mice were purchased from the Department of Laboratory Animal Science of Peking University (SCXK2014‐0004). All animals were housed in a pathogen‐free environment, under a 12‐hr dark/light cycle at 23 ± 1°C, 50%–60% humidity and allowed food and water ad libitum. All animal protocols were conducted in compliance with the national “Laboratory Animals Regulations” and “Fujian Regulations of Laboratory Animal Management” and approved by the Committee of Experimental Animal Care of Fujian Medical University (Fuzhou, Fujian, China).

Both SAMR1 and SAMP8 mice were randomly divided into two groups: the control and TMAO groups (*n* = 12, each group), according to a random number table produced by a computer. The groups were SAMR1‐control (R1‐C), SAMR1‐TMAO (R1‐T), SAMP8‐control (P8‐C) and SAMP8‐TMAO (P8‐T). Mice in the TMAO treatment groups were given TMAO (Sigma, USA) dissolved in water at a concentration of 1.5% for 16 weeks, while mice in control groups were supplied with sterile water.

### 4.4 Behavioural tests

At the end of TMAO treatment, all mice underwent behavioural tests, including the Y‐maze test and Morris water maze test (Yang et al., 2014; Zhang, He, Chen, Wang & Ma, 2008). Detailed methods are provided in the Data S1.

### 4.5 Measurement of plasma TMAO in mice

At the end of the behavioural tests, the animals were weighed and anaesthetized with 1% pentobarbital sodium (30 mg/kg, intraperitoneally) (Tang et al., 2013). Blood samples were collected for TMAO measurement. The procedures of TMAO measurement were undergone as described previously.

### 4.6 Tissue preparation

Detailed methods are provided in the Data S1.

### 4.7 Transmission electron microscope (TEM) examination

Detailed methods are provided in the Data S1 (Yang et al., 2014).

### 4.8 Senescent cell double staining with associated β‐galactosidase and immunohistochemistry

Detailed methods are provided in the Data S1 (Geng et al., 2010; Lin, Hong, Zou & Chen, 2015; Zhang et al., 2014).

### 4.9 Immunohistochemical staining

Detailed methods are provided in the Data S1 (Lin et al., 2015; Zhang et al., 2014).

### 4.10 Western blotting

Detailed methods are provided in the Data S1 (Lin et al., 2015).

### 4.11 Total SOD activity measurements

Detailed methods are provided in the Data S1 (Song et al., 2014).

### 4.12 Hydrogen peroxide measurements

Detailed methods are provided in the Data S1 (Begieneman et al., 2016).

### 4.13 Quantitative real‐time PCR (qRT–PCR)

Detailed methods are provided in the Data S1 (Lin et al., 2015).

### 4.14 Statistical analysis

Data from all experiments are expressed as the mean ± *SD* or mean ± SEM. All statistical analyses were performed using spss 18.0 software package, and *p* values below 0.05 were considered significant. Prism GraphPad software was used to generate the diagrams. The level of plasma TMAO in humans was analysed using one‐way ANOVA, and comparisons between two groups were performed using Tukey's multiple comparison test. In behavioural tests, data from escape latency and speed in the Morris water maze test were analysed with a repeated‐measures two‐way analysis of variance. All other data acquired from behavioural tests and other tests were analysed using two‐way ANOVA, and comparisons between two groups were performed using Tukey's multiple comparison test.

## CONFLICT OF INTEREST

None declared.

## AUTHOR CONTRIBUTIONS

HH, LZ and DL designed the experiments and conducted the study. DL wrote the manuscript. HH, YK and RZ revised the manuscript. LJ, SC and BP helped to revise the manuscript. CL, MZ and YK performed plasma TMAO measurement. AZ and XS performed animal management, TMAO treatment and behavioural tests. DL and AZ performed TEM examination, senescent cell double staining, immunohistochemical staining, Western blotting, total SOD activity and hydrogen peroxide measurements, and qRT–PCR. DL and YK performed statistical analysis. HH and LZ are the guarantors of this work and, as such, have full access to all the data in the study and take responsibility for the integrity of the data and the accuracy of the data analysis.
