# Supplementary material for: Trimethylamine‐N‐oxide promotes brain aging and cognitive impairment in mice
Source: Aging Cell. 2018 May 10;17(4):e12768. doi: 10.1111/acel.12768 (PMC6052480; doi:10.1111/acel.12768)
Supplement: Supplementary file 1 [file ACEL-17-na-s001.docx]

**Supporting information**

**Materials and methods**

**Behavioural tests (Zhang *et al*. 2008; Yang *et al*. 2014).**

At the end of TMAO treatment, all mice underwent behavioural tests, including the Y-maze test and Morris water maze test. The Y-maze had three arms with an angle of 120º between each arm and was made of Plexiglas. Each arm was 30 cm long × 8 cm wide × 15 cm high and covered with white paper. Obvious makers were placed on the walls of each arm. The arm of the maze was sprayed with alcohol after each individual trial to eliminate olfactory stimuli. Spontaneous alternation performance was performed as described previously. Generally, each mouse was placed on the end of one arm and allowed to explore the apparatus for 5 minutes. The total number of arms entered and the sequence made by the mouse was recorded after each trial. The total activity of the mouse was determined by the total number of arms entered; however, the effective alternation opportunity was calculated by subtracting 2 from the total number of arm entries. Percentage alternation was calculated by dividing the number of trials containing entries into all three arms by the alternation opportunity × 100. Novel object recognition tests involved two trials separated by an inter-trial interval. The three arms were randomly devised: the start arm, in which the mouse began to explore (always open); the novel arm: which was closed at the first trial but opened at the second trial: and the other arm (always open). The first trial lasted 10 minutes, in which the mouse was allowed to explore the start arm and the other arm, while the novel arm was closed. After a 4-hour inter-trial interval, the second trial was carried out. The mouse was placed back in the apparatus in the start arm as before, with a free approach to the three arms for 5 minutes. The time spent in each arm and the total number of entries were recorded and analysed. Performance of each mouse in all three arms was calculated as percentage during the 5 minutes of the trial.

The Morris water maze is a black circular tank filled with water (24±1℃), which was 120 cm in diameter and 30 cm in height. The maze was divided into 4 equal quadrants. A hidden escape platform (diameter: 10 cm, height: 23 cm) submerged 1.0 cm below the surface of the water was placed in the middle of one of the quadrants. MWM performance was tested as described previously. Generally, each mouse completed four trials per day with at least a 10-minute inter-trial interval for 6 days, which was called the acquisition trial phase. Four start points serving as the water entry position were equal to the hidden escape platform in length. Each mouse was permitted to find the goal within 60 seconds. If an animal was not able to find the platform within 60 s, it was gently guided to the platform and allowed to stay there for 15 seconds. The time taken to locate the goal within 60 seconds was equivalent to the escape latency. Escape latency and the average speed to get to the platform were recorded for each trial (n=6 per group). On the seventh day, a “probe test” was carried out to evaluate the strength of spatial memory retention. The mouse was allowed to explore the maze freely without the platform for 60 seconds. Two indexes were recorded: the amount of time spent in the target quadrant in which the platform was located previously and the number of times when a mouse exactly crossed over the previous position of the platform. Behavioural parameters were tracked and analysed.

**Tissue preparation.**

Animals were weighed and anaesthetised with 1% pentobarbital sodium (30 mg/kg, intraperitoneally). After blood collection, the mice were transcardially perfused with 0.1 mol/L phosphate-buffered saline (PBS; 15 mL). The brains were rapidly removed, placed on ice and sagittally cut into two halves. One side of the cerebral hemisphere was prepared for transmission electron microscopic examination, immunohistochemical staining and senescence-associated beta-galactosidase (SA-beta-GAL) staining. For TEM examination, the hippocampal CA1 region was dissected out quickly and was fixed by immersion in 2.5% buffered glutaraldehyde. For both SA-beta-GAL and immunohistochemical staining, brains were treated with 4% paraformaldehyde for 4 hours and then transferred to 20% sucrose at 4°C overnight (16 h) and subsequently to 30% sucrose. Samples were cut into 40 μm slices on a freezing microtome (Leica CM1850, Germany) and stored at −20°C in a cryoprotectant solution, which was mixed of 30% glycerine, 30% ethylene glycol and 40% 0.1 M phosphate-buffered saline (n=6, each group). The other side of the cerebral hemisphere was quickly frozen in liquid nitrogen, then stored at -80℃ and prepared for Western blotting and oxidative stress analysis.

**Transmission electron microscope (TEM) examination (**[**Yang *et al.* 2014**](#_ENREF_48)**)**

After tissue preparation for TEM examination, tissue blocks were post-fixed in 1% osmium tetroxide, dehydrated in ascending grades of ethanol, embedded in epoxy resin, and examined with TEM (n=6, each group).

**Senescent cell double staining with associated β-galactosidase (**[**Geng *et al.* 2010**](#_ENREF_15)**) and immunohistochemistry (**[**Zhang *et al.* 2014**](#_ENREF_50)**;** [**Lin *et al.* 2015**](#_ENREF_27)**).**

For SA-beta-GAL staining, frozen sections were washed 6 times in PBS and fixed and stained in a freshly prepared SA-beta-GAL staining solution containing 1 mg/mL of 5-bromo-4-chloro-3-indolyl-/3-D-galactopyranoside (X-Gal), 40 mmol/L citric acid, 100 mmol/L sodium phosphate, 5 mmol/L potassium ferrocyanide, 5 mmol/L potassium ferricyanide, 150 mmol/L NaCl, and 2 mmol/L MgCl_2_, at pH 6.0, as described previously ([Geng *et al.* 2010](#_ENREF_15)). After incubation in the dark at 37°C for 16 hours, sections were washed and counterstained with nuclear fast red.

To identify the senescent cells, we used immunohistochemical staining after SA-beta-GAL staining. After washes in PBS, sections were treated with 3% hydrogen peroxide for 10 minutes to quench endogenous peroxidase and then washed in PBS. Then, sections were blocked for nonspecific binding of antibody with 5% normal goat serum for 30 minutes at room temperature, followed by incubation with primary antibodies at 4°C overnight. The following primary antibodies were used: rabbit monoclonal anti-NeuN antibody (a marker of neurons, Abcam, 1:2000), mouse monoclonal anti-GFAP antibody (a marker of astrocytes, Millipore, 1:2000) and mouse monoclonal anti-Iba1/AIF1 antibody (a marker of microglia, Millipore, 1:2000). PBS was used as a blank control. Then, samples were incubated with horseradish peroxidase (HRP)-conjugated goat anti-rabbit antibody or HRP-conjugated goat anti-mouse antibody (1:500, Millipore, USA) for 1 h at room temperature. The sections were washed thoroughly in PBS, mounted onto polylysine-coated glass slides, air dried, dehydrated in ethanol, cleared in xylene, and then cover-slipped with permanent mounting medium (Vector Laboratories). Then, sections were observed and photographed under a microscope (Nikon, Japan).

**Immunohistochemical staining (**[**Zhang *et al.* 2014**](#_ENREF_50)**;** [**Lin *et al.* 2015**](#_ENREF_27)**).**

The procedures were performed as described previously. Sections were incubated with primary antibodies at 4°C overnight. The following primary antibodies were used: rabbit monoclonal anti-synaptophysin antibody (Abcam, 1:500), rabbit polyclonal anti-PSD-95 antibody (Abcam, 1:500) and rabbit monoclonal anti-NMDAR1 antibody (Abcam, 1:500).

**Western blotting (**[**Lin *et al.* 2015**](#_ENREF_27)**).**

The protein was collected from the whole hippocampus of the mice in cold lysis buffer with protease inhibitors (0.1 M phosphate-buffered saline, 1% Triton X-100, 2.5 mM Na_4_P_2_O_5_·10H_2_O, 2 mM NaF, 1% protease inhibitor cocktail). After 10 minutes on ice, the samples were centrifuged at 16,000 g for 15 minutes at 4℃, and the supernatant was extracted. After total protein concentrations were measured, the samples were mixed with lysis buffer and 6× sample buffer (125 mM Tris, pH 6.8, 10% glycerol, 10% sodium dodecyl sulphate, 130 mM dithiothreitol, and 0.006% bromophenol blue). Then, the protein was boiled for 10 minutes and stored at -20℃ before use. A total of 30 μg protein was separated by sodium dodecyl sulphate-polyacrylamide gel electrophoresis and transferred onto polyvinylidene difluoride membrane. After blocking with 5% nonfat dry milk solution in 0.1% TBST for 1-1.5 h at room temperature, the membrane was cut into several pieces according to the molecular weight of each target protein. Then, the membrane pieces were incubated overnight at 4°C with different primary antibodies. The following primary antibodies were used: mouse polyclonal anti-β-actin (Cell Signaling Technology, 1:1000), rabbit monoclonal anti-synaptophysin antibody (Abcam, 1:2000), rabbit polyclonal anti-PSD-95 antibody (Abcam, 1:1000), rabbit monoclonal anti-NMDAR1 antibody (Abcam, 1:1000), rabbit polyclonal anti-mTOR antibody (Cell Signaling Technology, 1:1000), rabbit polyclonal anti-phosphor-mTOR antibody (Ser2481; Cell Signaling Technology,1:1000), rabbit monoclonal anti-p70s6k antibody (Cell Signaling Technology, 1:1000), rabbit monoclonal anti-phosphor-p70s6k antibody (Thr389; Cell Signaling Technology, 1:1000), rabbit polyclonal anti-4EBP2 antibody (Cell Signaling Technology, 1:500) and rabbit monoclonal anti-phosphor-4EBP1 antibody (Thr37/46; Cell Signaling Technology, 1:500). After the samples were washed with 0.1% TBST five times, the membranes were incubated for 90 minutes at room temperature with horseradish peroxidase-coupled secondary antibodies (Cell Signaling Technology, 1:3000). After they were washed three times with TBST the membrane bands were detected with an ECL kit (Millipore) and visualised by exposure to Kodak film. The density of protein bands was quantified with ImageJ software, and the expression of each target protein was calculated as a relative value to the levels of β-actin.

**Total SOD activity measurements (**[**Song *et al.* 2014**](#_ENREF_38)**).**

Total SOD activity was determined with an OxiSelect Superoxide Dismutase Activity Assay (Cell Biolabs, Inc.) according to the manufacturer’s instructions. This kit uses a xanthine/xanthine oxidase system to generate superoxide anions. The chromagen produces a water-soluble formazan dye upon reduction by superoxide anions. The activity of SOD is determined as the inhibition of chromagen reduction. Briefly, tissue lysate supernatant, xanthine solution, chromagen solution, 10×SOD assay buffer, and water were mixed and added into a 96-well microtiter plate. Then, 10 µL prediluted 1× xanthine oxidase solution was added into each well and incubated for 1 hour at 37°C. The absorbance was read at 490 nm on a microplate reader. The SOD activity was calculated according to the standard curve.

**Hydrogen peroxide measurements (**[**Begieneman *et al.* 2016**](#_ENREF_5)**).**

The presence of hydrogen peroxide was determined using the Hydrogen Peroxide Assay Kit (Cell Biolabs Inc.), according to the manufacturer’s instructions. All reagents were prepared and mixed thoroughly before use. The hydrogen peroxide standards were prepared simultaneously with the samples so they could be assayed together. Each sample, including unknown and standard samples, were assayed in duplicate or triplicate. Then, 90 µL of standard or sample was added to a microcentrifuge tube, followed by 10 µL of the 10 mM TCEP solution (two 1.5 mL vials of a 10 mM Tris (2-carboxyethyl) phosphine solution in methanol). The tubes were vortexed thoroughly and incubated for 30 minutes at room temperature. Then, 25 µL of the TCEP-treated standard or sample was transferred to fresh microcentrifuge tubes. Next, 250 µL of Lipid Working Reagent was added to each tube. The contents were vortexed thoroughly and incubated on a shaker for 30 minutes at room temperature. The sample and standard tubes were centrifuged at 12,000 x g for 5 minutes to remove any precipitate. The sample supernatant or standard (~275 µL) was transferred to a microtiter plate. The plate was read at 540-600 nm (595 nm optimal). The concentration of hydrogen peroxide was calculated in the samples by comparing the sample absorbance to the standard curve.

**Quantitative real-time PCR (qRT-PCR) (**[**Lin *et al.* 2015**](#_ENREF_27)**).**

Total RNA was extracted using TRIzol reagent (Gibco-BRL; Life Technologies, Carlsbad, CA), according to the manufacturer’s instructions. Complementary DNA (cDNA) was synthesised using a reverse transcription kit provided by TaKaRa Bio Inc. (Shiga, Japan). The mRNA expression levels of GAPDH, mTOR, p70s6k and 4EBP2 were quantified by SYBR Green-based quantitative real-time PCR using a SuperReal PreMix Plus (SYBR Green) kit (Tiangen, Beijing, China). △cycle threshold (Ct) = (test gene) Ct - (GAPDH) Ct; △Ct of SAMR1-Control and △Ct of SAMP8-Control were considered as the baseline respectively, and △△Ct = (test gene) △Ct - (baseline) △Ct; fold change =2 ^-△△Ct^. The primers for GAPDH, mTOR, p70s6k and 4EBP2 are listed in Supplemental Table S2.

**Table S1 Baseline characteristics of subjects**

Data are shown as the mean ± SD (one-way ANOVA followed by Tukey post hoc test. BMI=Body Mass Index, SBP: systolic blood pressure, DBP: diastolic blood pressure, TC: total cholesterol, TG: triglyceride, LDL-C: low density lipoprotein cholesterol, HDL-C: high density lipoprotein cholesterol, ALT: glutamic-pyruvic transaminase, AST: glutamic oxalacetic transaminase, FBG: fasting blood-glucose, Y: young adults (n=168), M: middle-aged adults (n=118), E: elderly adults (n=141).

**Table S2 Primer sequence list**

| Gene Upstream primer(5’-3’) Downstream primer(5’-3’) |
| --- |
| mTOR CATCACCAATGCCACCACTG AGGAGATAGAACGGAAGAAGCC  4EBP2 GTTGGACCGTCGCAATTCTC CCTCCTTCCACATGGCAGTAG  p70s6k GCCTCCCTACCTCACACAAG ACTCACATCCTCTTCAGATTGC  GAPDH ACCACAGTCCATGCCATCAC TCCACCACCCTGTTGCTGTA |


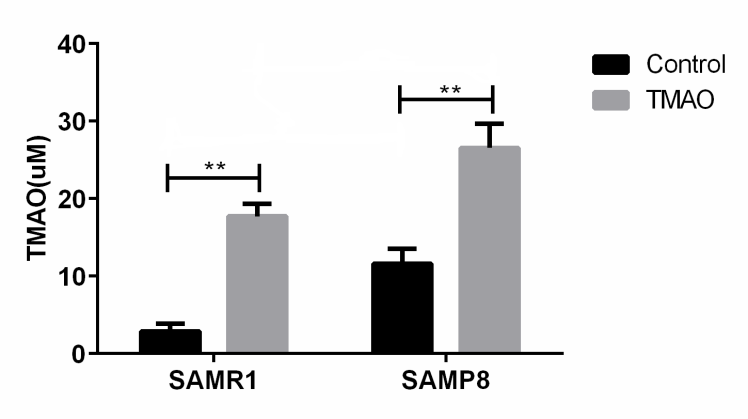


Fig. S1. Circulating TMAO levels increase after TMAO treatment in both SAMR1 and SAMP8 mice.


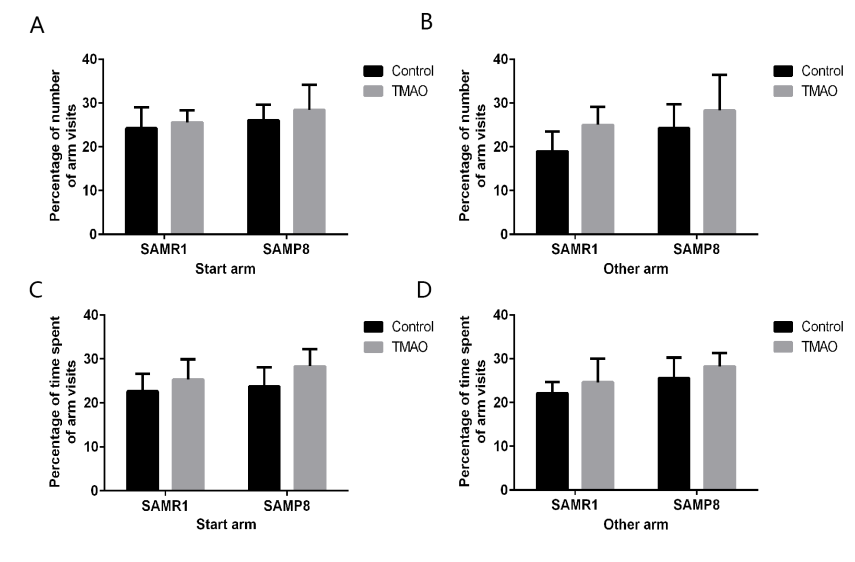


Fig. S2 Data acquired from behavioral tests.


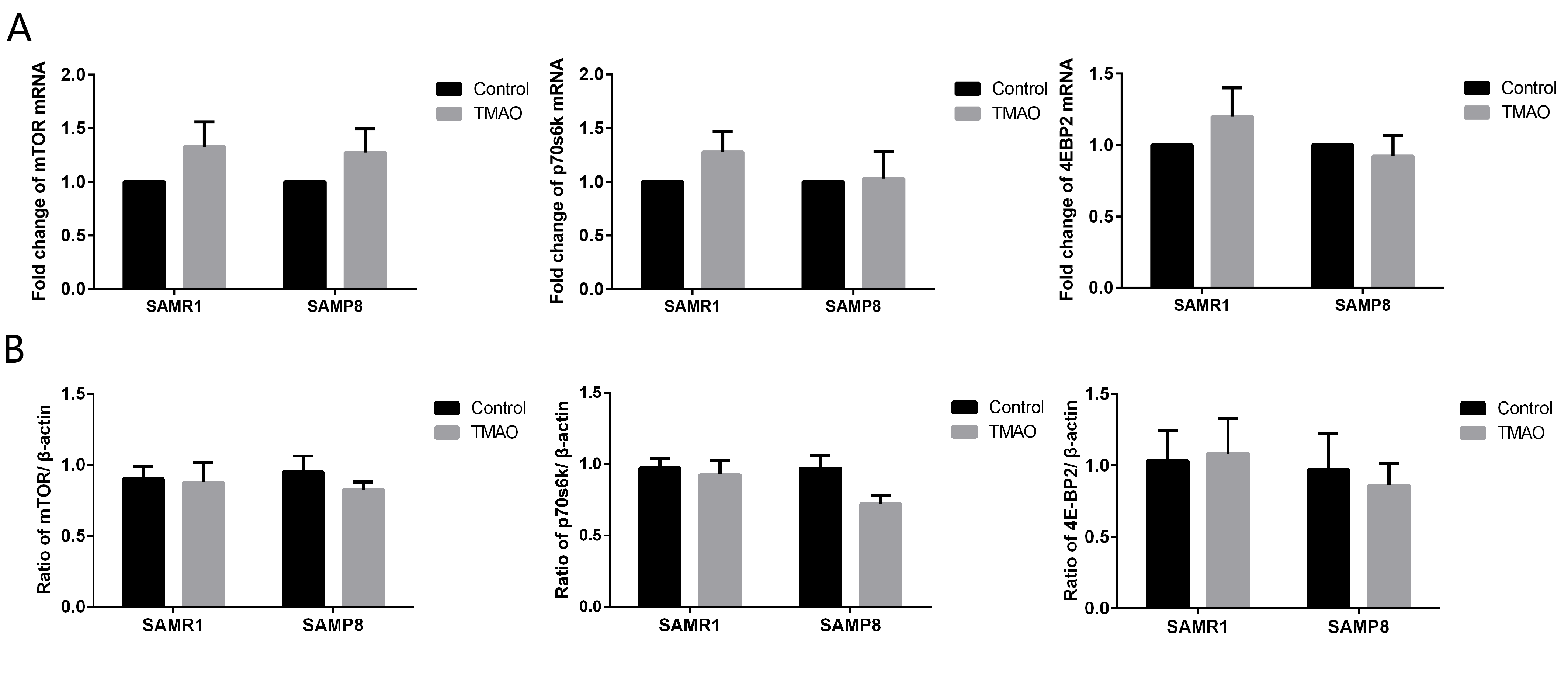


Fig. S3 Fold-changes of mRNA expression and total protein expression of the mTOR signaling pathway in the hippocampus.


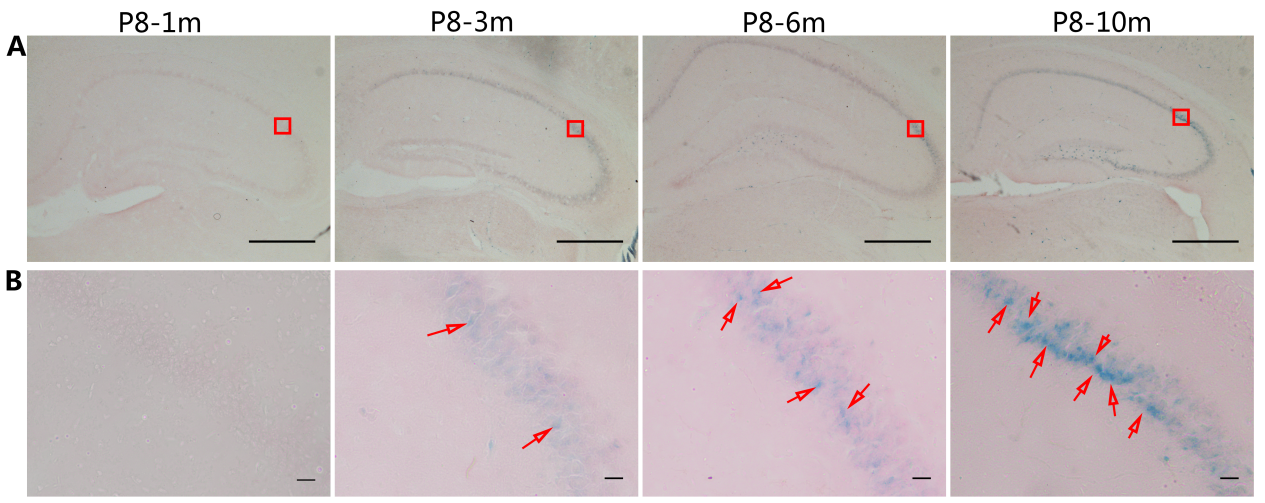


Fig. S4 SA-beta-GAL staining in the hippocampus of different aged SAMP8 mice.


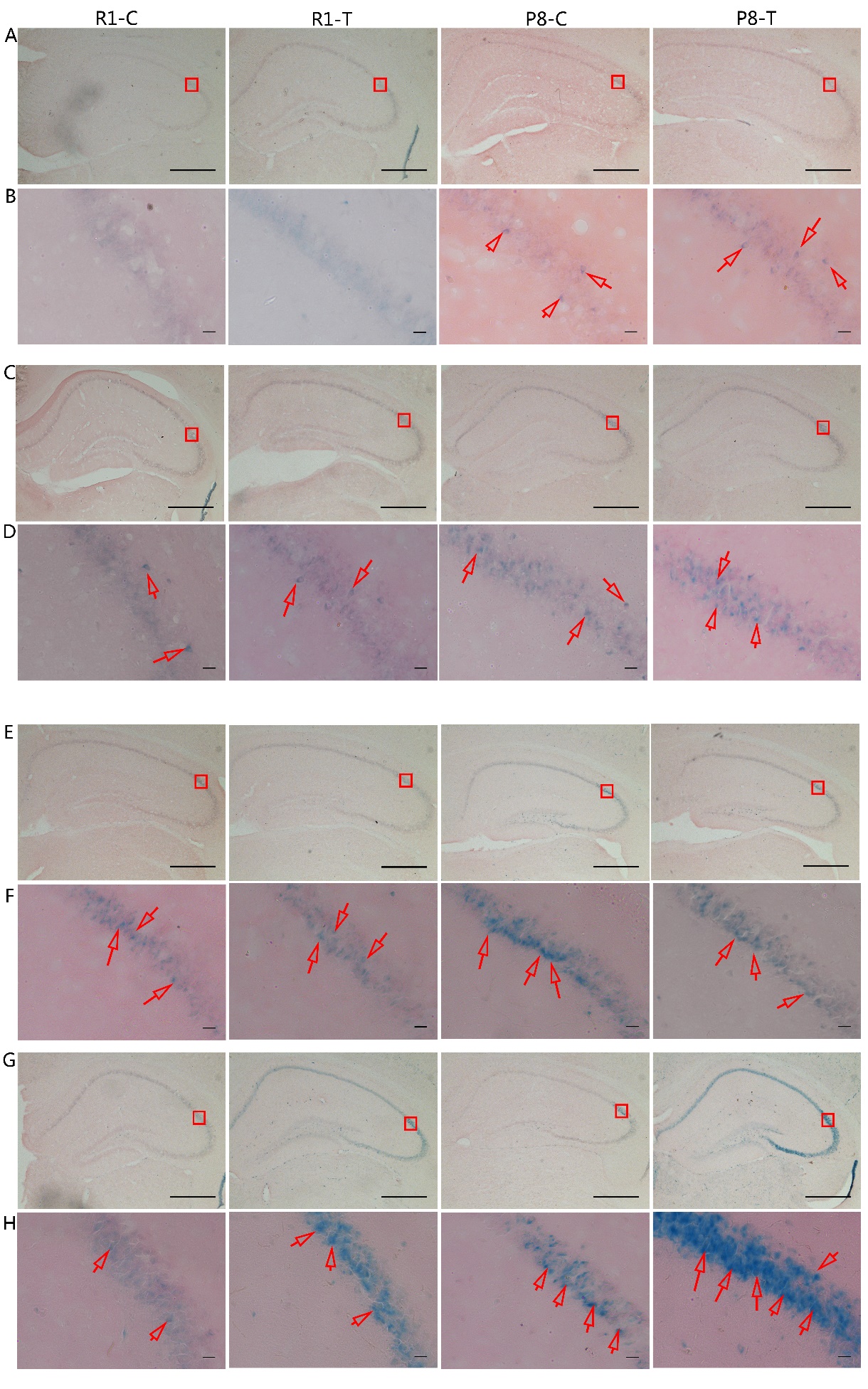


Fig. S5 SA-beta-GAL staining in the hippocampus of different TMAO treatment in SAMR1 and SAMP8 mice.

**Figure legends**

Fig. S1. The circulating TMAO levels were quantified using stable isotope dilution liquid chromatography with mass spectrometry (LC/MS/MS) in both SAMR1 and SAMP8 mice after TMAO treatment. Data are shown as the mean ± SD, n=12 each group, two-way ANOVA followed by a Tukey post hoc test; ** p<0.01.

Fig. S2. In novel object recognition, the percentage of number of visits and the time spent obtained from the start arm and other arm respectively were not significantly different. Data are shown as the mean ± SEM. Data were analysed using two-way ANOVA, and comparisons between two groups were performed using Tukey's multiple comparison test. n=12 each group..

Fig. S3. (*A*) The mRNA levels of mTOR, p70s6k and 4EBP2 were not significantly different in the groups assessed by RT-PCR. In addition, (*B*) the expressions of proteins (mTOR, p70s6k,and 4EBP2) were not significantly different in the groups monitored by Western blotting. β-actin was used as a loading control. The bands in the Western blotting were scanned, and the ratios of optical density of specific bands and β-actin are illustrated. Data are shown as the mean ± SEM (n=6 each group, two-way ANOVA, comparisons between two groups were made using Tukey's multiple comparison test). The samples derived from the same experiment and the blots were processed in parallel.

Fig. S4. SA-beta-GAL was detected in the hippocampal CA3 region of different aged SAMP8 mice. The blue cells indicated SA-beta-GAL-positive cells. These results showed that the number of senescent cells increased in the hippocampal CA3 region during the aging process. (n=6 each group, scale bar: A=50 μm, B=100 μm)

Fig. S5. SA-beta-GAL was detected in the hippocampal CA3 region of different TMAO treatments in both SAMR1 and SAMP8 mice. The blue cells indicated SA-beta-GAL-positive cells. (*A-D*) There was no significant difference in the number of senescent cells between the 0.75% TMAO treatment group and control group, and between the 1.5% TMAO treatment group and control group in both SAMR1 and SAMP8 mice after 8 weeks. (*E and F*) There was no significant difference in the number of senescent cells between the 0.75% TMAO treatment group and control group in both SAMR1 and SAMP8 mice after 16 weeks. (G and H) Compared with the control groups respectively, the number of senescent cells increased significantly in the 1.5% TMAO groups in both SAMR1 and SAMP8 mice after 16 weeks. (n=6 each group, scale bar: A, C, E, G=50 μm, B, D, F, H=100 μm)
